# Correlation Analysis of Vestibular Symptoms and Migraine and Non-migraine Headaches: An Epidemiological Survey of 708 Female Nurses

**DOI:** 10.3389/fnins.2022.925095

**Published:** 2022-06-30

**Authors:** Tongxiang Diao, Jinling Zhu, Lisheng Yu, Xin Ma

**Affiliations:** Department of Otolaryngology, Head and Neck Surgery, People’s Hospital, Peking University, Beijing, China

**Keywords:** vestibular symptoms, dizziness, vertigo, headache, migraine, nurses

## Abstract

**Objective:**

This study is oriented to study the correlation between different vestibular symptoms and migraine and non-migraine headaches.

**Materials and Methods:**

A questionnaire containing factors related to vestibular symptoms and migraine was designed to survey nurses in a tertiary hospital. Then, all study subjects were divided into three groups: no headache, migraine, and non-migraine headache, and the general physical condition and incidence of different vestibular symptoms were compared among the three groups.

**Results:**

Among all the 708 subjects, 233 had headaches. The incidence of migraine was 13.3%. There were 235 cases had vestibular symptoms. Dizziness and vertigo are independent factors related to headaches, especially migraine. The risk of migraine and other types of headaches in the vertigo group is 2.808 and 2.526 times of those without vertigo, while in the dizziness group, the risk is 8.248 and 5.732 times of those without dizziness.

**Conclusion:**

Different vestibular symptoms were all related to migraine. And different vestibular symptoms and non-migraine headaches also showed a clear correlation.

## Introduction

In 2012, the Bárány Society first proposed and formulated the definition and diagnostic criteria of vestibular migraine and probable vestibular migraine (VM) (Lempert et al., 2012), in which, the vestibular symptoms are defined as spontaneous vertigo including internal vertigo, external vertigo, positional vertigo, visually induced vertigo, head motion-induced vertigo, and head motion-induced dizziness with nausea (the dizziness here is only referred to a sense of spatial disorientation). Since then, the correlation between vestibular symptoms and headache has attracted much more attention from clinicians. At present, the vestibular symptoms included in the diagnostic criteria for VM are mainly vertigo and dizziness accompanied by spatial disorientation.

Meanwhile, the 2009 International Classification of Vestibular Diseases defines vestibular symptoms as vertigo, dizziness, vestibulo-visual, and postural symptoms (Bisdorff et al., 2009). A few studies have already proposed that dizziness and postural symptoms are also significantly related to migraine besides vertigo (Vuković et al., 2007). Moreover, although the relationship between vestibular symptoms and migraine has gradually become clear, few studies have focused on the relationship between vestibular symptoms and non-migraine headaches. The nursing profession is subject to occupational stress, which can be a trigger for headaches. Durham et al. (1998) found that 17% of nurses have migraines according to the International Headache Society (IHS) criteria. Menon and Remadevi (2021) also found that a total of 20% of nursing students had headaches of which 85% had migraine in their study. In this study, nurses who have a high incidence of headaches were selected as the subjects (Wang et al., 2015), to further explore the correlation between different vestibular symptoms and migraine and non-migraine headaches, providing more clinical diagnostic evidence for VM.

## Materials and Methods

### Study Design

This is a cross-sectional study conducted through a self-designed questionnaire among the nurses from a tertiary A hospital in Beijing. Researchers have undergone uniform training to be familiar with the contents of the questionnaire. The electronic questionnaire was distributed through the WeChat platform. Before distributing the questionnaire, researchers went to each department to conduct training for filling the questionnaire, clarifying the purpose, significance, and filling requirements of the survey to ensure its quality. The questionnaires are filled out voluntarily by the respondents. It takes about 20 min to complete each questionnaire. Complete and effective questionnaires are selected by researchers in the end.

### Participants

**FIGURE 1:**
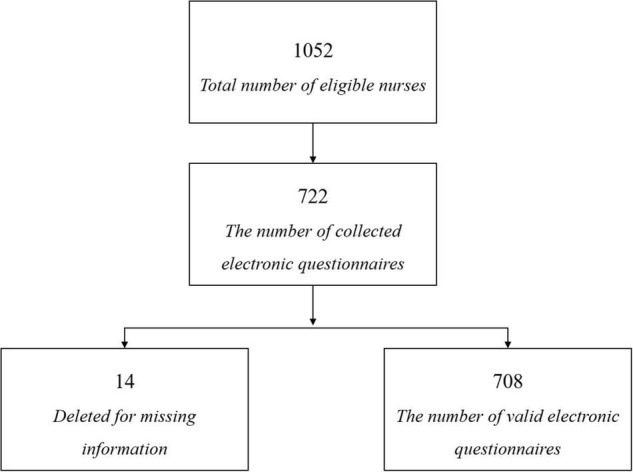
The flowchart.

The nurses were on-the-job from January to June 2019. Inclusion criteria were as follows: (1) Registered nurses in tertiary A hospitals; (2) Female; (3) Engaged in ward nursing work with working experience no less than 3 years; (4) Can answer questions independently; (5) Volunteer to participate in the survey; (6) Vestibular dysfunctions such as Meniere’s disease and vestibular neuritis were excluded; (7) Organic lesions, such as acoustic neuroma, were excluded. Exclusion criteria were as follows: severe physical and mental illness and those who cannot complete the survey due to various reasons. A total of 1,052 questionnaires were distributed by the WeChat platform, 722 were recovered, and finally, 708 valid questionnaires were collected with 14 questionnaires deleted for missing Information (Figure 1).

### Data Collection

This study used a self-designed questionnaire, including the contents as following:

#### General Physical Condition

Age, department (internal medicine, surgery), professional title (junior, middle-level, senior), BMI, whether to work the night shift, addiction to tobacco or alcohol (yes or no), and exercise status. The exercise status includes the following: (1) Daily pedometer steps (<2,000, 2,000–5,000, 5,000–10,000, and >10,000); (2) Is it possible to guarantee moderate-intensity exercises more than 30 min at least twice a week? Chronic disease history includes the following: hypertension, hyperglycemia, hyperlipidemia, ototoxic drug, noise exposure which refers to an 8 h average exposure of greater than 83 dBA (Roberts and Neitzel, 2019), and long-term low-dose noise exposure (limited or similar to Walkman, more than 60 min, and/or more than 4 times a week).

#### The Clinical Characteristics of Headache

Include the following: (1) headache history and (2) the modified ID-migraine: ① Your headaches limited your ability to work, study, or do what you needed to do for at least one day (Yes or No). ② You felt nauseated or sick to your stomach (Yes or No). ③ Light bothered you a lot more than when you do not have headaches (Yes or No). When ID-migraine was initially proposed, it only required to record headaches during the last 3 months. Individuals who indicated that they had two or three of these features were said to screen positive for migraine (Rapoport and Bigal, 2004). However, this study considered that migraine is a paroxysmal disease, and the frequency of attacks is variable, so the 3-month time limit was canceled. Clinical characteristics also include (3) the location, nature, and duration of the headache.

#### The Clinical Characteristics of Vestibular Symptoms

Including (1) history of vestibular symptoms and (2) classification of vestibular symptoms, such as vertigo, dizziness, and postural symptoms. These three vestibular symptoms are defined as follows: vertigo is the sensation of self-motion when no self-motion is occurring or the sensation of distorted self-motion during an otherwise normal head movement; dizziness is the sensation of disturbed or impaired spatial orientation without a false or distorted sense of motion; postural symptoms are balance symptoms related to maintenance of postural stability, occurring only while upright (seated, standing, or walking) (Bisdorff et al., 2009); (3) if vertigo is combined, the frequency of vertigo attacks, duration, incentives, and mitigating factors are registered.

### Ethics Statement

The Peking University People’s Hospital Ethical permission committee approved the study (2019PHB099-01) and all subjects provided their informed consent.

### Statistical Analysis

SPSS 23.0 statistical software was used for statistical analysis. Frequency and percentage are used to describe the distribution, while the means are used to describe the average level. The Chi-square test and ANOVA are used to compare categorical and continuous variables, respectively. According to whether migraine was combined, all participants were divided into no headache group, migraine group, and non-migraine headache group. First, univariate analysis was used to screen statistically significant influencing factors, then multivariate analysis was used to explore the independent correlation factors between migraine and other types of headaches. All *p* values are bidirectional, and *p* < 0.05 values are considered statistically significant.

## Results

### Epidemiological Characteristics

A total of 708 women nurses were enrolled in this study, with an average age of 32.39 ± 8.924 years old (23–57 years old), and an average BMI of 21.667 ± 3.08 (13.521–30.819). Among the nurses, 454 were in internal medicine and 254 were in surgery. Junior, middle level, and senior professional titles are 342, 225, and 141, respectively. The general physical condition was well, subjects with high blood pressure, diabetes, and hyperlipidemia were 53, 26, and 45, respectively. Subjects with smoking and drinking history were 4 and 15, respectively. The specific description of its epidemiological characteristics is shown in Table 1.

**TABLE 1:** Epidemiological characteristics of 708 nurses.

Variables		$\bar{\text{x}}\pm$ μ	*n*
Age		32.39 ± 8.924	708/708
Department	Internal medicine		454/708 (63.0%)
	Surgery		254/708 (35.2%)
Professional title	Junior		342/708 (48.3%)
	Middle-level		225/708 (31.8%)
	Senior		141/708 (19.9%)
BMI		21.667 ± 3.080	422/708 (59.6%)
Night shift			469/708 (66.2%)
Pedometer steps (/day)	<2000		264/708 (37.3%)
	(2000, 5000)		33/708 (4.7%)
	(5000, 10000)		215/708 (30.4%)
	≥10000		196/708 (27.7%)
Moderate-intensity exercise over 30 min at least twice a week			182/708 (25.7%)
Hypertension*			53/708 (7.5%)
Diabetes*			26/708 (3.7%)
Hyperlipidemia*			45/708 (6.4%)
Ototoxic drug use history*			6/708 (0.8%)
Noise exposure*			29/708 (4.1%)
Long-term low-dose noise exposure			172/708 (24.3%)
Smoking history*			4/708 (0.6%)
Drinking history*			15/708 (2.1%)

PS: (1) The data on BMI is incomplete, with an average of 21.67 (13.521∼30.819), which is not included in the follow-up statistics; (2) The positive response rate of history of hypertension, diabetes, hyperlipidemia, ototoxic drugs, noise exposure, and addiction to tobacco or alcohol are less than 10%, which are not included in the following statistics. *Indicates that the response rate is <10%.

### Clinical Characteristics of Headaches

**FIGURE 2:**
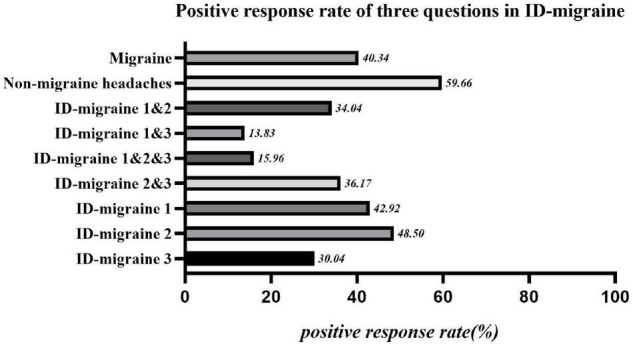
Positive response rate of three questions in ID-migraine. ID-migraine 1: If there is one day or more you can’t work, study, or do daily activities normally because of headache?; ID-migraine 2: Is there nausea or stomach upset during headache?; ID-migraine 3: Is there photophobia companied with headache?

Among all the 708 subjects, 233 (28.7%) had headaches, with an average history of 7.91 ± 7.642 years, of which 94 cases were consistent with the diagnosis of migraine (individuals who indicated that they had two or three of these features were said to screen positive for migraine), accounting for about 13.3% of all subjects, and 40.3% of headache population. According to whether migraine was companied, headache patients were divided into migraine group (94 cases) and non-migraine headache group (139 cases) and included in the follow-up statistical analysis. The positive response rate of each question in ID-migraine is shown in Figure 2.

### Characteristics of the Vestibular Symptoms

**FIGURE 3:**
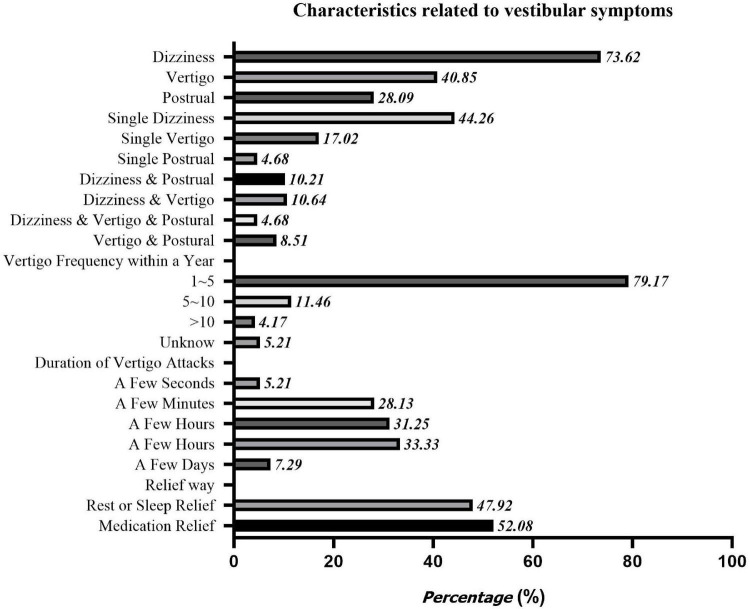
Characteristics related to vestibular symptoms.

Among all the 708 subjects, 235 (33.2%) complained of multiple vestibular symptoms, with an average history of 5.91 ± 6.551 years. Among them, 155 cases had only one single vestibular symptom, and 80 cases had multiple vestibular symptoms. According to the 2019 Bárány Association’s classification criteria for vestibular symptoms, this study divided vestibular symptoms into vertigo, dizziness, and postural symptoms, of which 96 cases were combined with vertigo, 173 cases were combined with dizziness, and 66 cases were combined with postural symptoms. The clinical characteristics of the vestibular symptoms are shown in Figure 3.

### Correlation of Different Vestibular Symptoms and Headaches

According to whether migraine is accompanied, all the subjects were divided into three groups: no headache, migraine, and non-migraine headache group. Univariate analysis was used to screen out the statistically significant factors: age, professional title, whether to work night shifts, dizziness, vertigo, and postural symptoms, then all these factors were included in the following multiple logistic regression. The results showed that: different vestibular symptoms all showed a certain correlation with migraine, among which dizziness and vertigo were independent related factors of migraine and non-migraine headaches. The incidence of combining migraine and non-migraine headaches were 2.808 and 2.526 times that in the non-vertigo group, respectively. The incidence of combining migraine and non-migraine headaches was 8.248 and 5.732 times that in the non-dizziness group, respectively (Table 2).

**TABLE 2:** Factors related to migraine and non-migraine headache.

A. Univariate analysis					
**Variables**		**Non-headache *(n = 475)***	**Migraine *(n = 94)***	**Non-migraine headache *(n = 139)***	***p***
Age		31.31 ± 8.586	33.72 ± 8.907	34.79 ± 9.541	0.000*
Department	Internal	326/475	65/94	105/139	0.288
	Surgery	149/475	29/94	34/139	
Professional title	Junior	249/475	41/94	55/139	0.000*
	Middle-level	155/475	26/94	42/139	
	Senior	71/475	27/94	42/139	
Night shift	No	145/475	29/94	67/139	0.000*
	Yes	330/475	65/94	72/139	
Pedometer steps (/day)	<2000	173/475	38/94	53/139	0.959
	(2000, 5000)	21/475	5/94	7/139	
	(5000, 10000)	146/475	25/94	44/139	
	≥10000	135/475	26/94	35/139	
Moderate intensity exercise over 30 min at least twice a week	No	356/475	66/94	104/139	0.623
	Yes	119/475	28/94	35/139	
Long-term low-dose noise exposure	No	351/475	72/94	114/139	0.142
	Yes	124/475	22/94	25/139	
Dizziness		54/475	54/94	65/139	0.000*
Vertigo		38/475	26/94	32/139	0.000*
Postural symptom		24/475	21/94	21/139	0.000*

**B. Multivariate analysis**					

**Variables**		**Migraine**		**Non-migraine**	
		**Odds ratio (95% CI)**	***p***	**Odds ratio (95% CI)**	***p***

Age		1.018 (0.975∼1.062)	0.420	1.015 (0.981∼1.050)	0.392
Professional title	Junior	1		1	
	Middle-level	0.880 (0.450∼1.720)	0.708	0.937 (0.536∼1.637)	0.818
	Senior	2.205 (0.864∼5.626)	0.098	1.421 (0.655∼3.080)	0.374
Night shift		1.875 (0.961∼3.658)	0.065	0.669 (0.406∼1.102)	0.114
Dizziness		8.248 (4.882∼13.933)	0.000*	5.732 (3.607∼9.110)	0.000*
Vertigo		2.808 (1.480∼5.328)	0.002*	2.526 (1.420∼4.492)	0.002*
Postural symptom		1.758 (0.834∼3.705)	0.138	1.200 (0.585∼2.463)	0.619

*P < 0.05.

## Discussion

### Incidence of Headaches and Migraine

Among all the 708 subjects admitted in this study, the incidence of headache and migraine were 28.7 and 13.3%, respectively. It has been reported that the incidence of idiopathic headaches in mainland China is about 23.8%, of which the incidence among women is around 36.8% (Yu et al., 2012). Research conducted by Shengyuan Yu found that the incidence of idiopathic headache, migraine, and tension-type headache among nursing staff with high pressure can be as high as 45.3, 14.8, and 26.2% (Wang et al., 2015). Compared with previous studies, the incidence of headaches in this study is lower, but the incidence of migraine is basically the same. We deem that the migraine is moderate to severe headache, which affects daily life and tends to be reported. While a mild headache, it tends to be thought of as a normal physiological phenomenon rather than a disease after a night shift. There is no undue fear of them, and people tend not to report it in the survey. This is also the reason for the gap between the electronic self-evaluation questionnaire and the face-to-face evaluation.

### The Correlation Between Different Vestibular Symptoms and Migraine

In this study, the incidence of dizziness, vertigo, and postural symptoms were 24.44, 13.56, and 9.32%, respectively. About 1/3 of the subjects had complained about vestibular symptoms, which was close to the lifetime prevalence of dizziness and balance instability among American adults (Formeister et al., 2018). Among all the nurses with vestibular symptoms, about 65.96% complained of a single vestibular symptom, and 34.03% complained of two or more vestibular symptoms, which reminded us that although the Barany Association distinguished dizziness, vertigo, vestibulo-visual and postural symptom clearly in 2009, different vestibular symptoms may coexist or appear sequentially in one patient (Bisdorff et al., 2009). The previous literature also pointed out that about 60% of patients with vestibular symptoms complained of multiple vestibular symptoms consistent with the result of our research (Kerber et al., 2017). Although most clinical researchers believe that only the patients with spontaneous vertigo that meets the current diagnostic criteria for VM can be diagnosed (Cohen and Escasena, 2015), there is a wide range of vestibular symptoms associated with migraine in fact. One study aimed at VM found that the descriptions of vestibular symptoms were complex and diverse, including instability (91%), dizziness (77%), vertigo (57%), internal head rotation (45%), or the feeling of being on a rocking boat (41%) (Cohen and Escasena, 2015). Migraine is also associated with a slight but significant postural instability originating from the central vestibule, which is manifested as a greater swing speed, a shift in the eccentric center of gravity, and an increase in stride length during tandem walking (Calhoun et al., 2011). In this study, different vestibular symptoms and migraine all showed a significant correlation. The dizziness group was more likely to be accompanied by migraine than the vertigo group (8.248 vs. 2.808), suggesting that we should not ignore the correlation between other vestibular symptoms and VM, to avoid a missing diagnosis of VM that may not meet the current diagnostic criteria (Yu et al., 2016).

### The Correlation Between Vestibular Symptoms and Non-migraine Headaches

We further found that different vestibular symptoms also correlated significantly with non-migraine headaches. Compared with vertigo group patients, the dizziness group patients were more likely to suffer from non-migraine headaches (5.732 vs. 2.526). These suggested that similar to migraine, non-migraine headaches could also be accompanied with vestibular symptoms. Migraine and tension-type headaches, the current most common primary headaches, were thought to be independent diseases according to the International Headache Classification [ICHD] (2018). However, there was a growing number of studies that had focused on the similarities between migraine and tension-type headache (TTH). In fact, Migraine patients often had typical TTH symptoms such as muscle tension and neck pain (Blaschek et al., 2012). Similarly, TTH patients often had symptoms of photophobia and phonophobia which could aggravate after activities (Spierings et al., 2001). In addition to the overlap of symptoms, patients with these two diseases could have similar pathogenic factors, such as age, sex, BMI, sleep disorders, negative emotions, sunshine exposure, anxiety, and depression (Wang et al., 2013; Tai et al., 2019). Migraine and TTH both had a significant familial predisposition, which could be reflected by the fact that the twins of affected individuals have obvious increased risks of migraine and TTH, confirming the importance of genetic factors in disease pathogenesis (Ligthart et al., 2018). Moreover, these two diseases are both thought to be related to central sensitization mechanisms (Yunus, 2007). The strict definitions of migraine and TTH in ICHD are artificial to some extent, and they may be one disease with different severities: TTH is mild, while migraine is severe (continuous severity theory; Waters, 1973) (Turner et al., 2015; Ligthart et al., 2018). The boundaries between them could become more blurred in young patients or in patients with chronic disease courses (Turner et al., 2015). In this study, the patients’ overall age was relatively young, and the relationships between migraine, non-migraine headache, and vestibular symptoms then showed high consistency.

## Limitation

Similar to several epidemiological studies, this study had some limitations that need to be addressed. The first was the self-misclassification of study subjects, including (1) patients were given certain options to describe their subjective symptoms, and had to choose from them; (2) patients were not provided with sufficient options which could cover all specific subcategories, leading to wrong classification of their symptoms. Second, the age distribution of study subjects is not regular, which may cause some bias in the results. Third, the view that vestibular dysfunction may induce visual impairment is not commonly accepted by patients, and we focused mainly on the correlations between vestibular symptoms and headache. Therefore, the vestibular visual symptoms were not included in this study. Finally, we did not provide relevant questions about the diagnostic criteria of tension headache. Although a great portion of non-migraine headaches might be tension headaches, without accurate diagnoses, we could not explore the relationship between dizziness and tension headaches, which will be further explored in our future studies.

## Conclusion

Different vestibular symptoms were all closely related to migraine, among which dizziness and vertigo were both significantly related. Compared with patients with vertigo, patients with dizziness were more likely to be accompanied by migraine. This suggested that when diagnosing vestibular migraine according to current criteria, some patients with non-vertigo vestibular symptoms might be missed. On the other hand, different vestibular symptoms also showed a clear correlation with non-migraine headaches.

## Data Availability Statement

The raw data supporting the conclusions of this article will be made available by the authors, without undue reservation.

## Ethics Statement

The studies involving human participants were reviewed and approved by The Peking University People’s Hospital Ethical Permission Committee (2019PHB099-01). The patients/participants provided their written informed consent to participate in this study.

## Author Contributions

XM, TD, and JZ supervised this research. XM and TD contributed to the analysis and interpretation of data and wrote the first draft of the manuscript. XM and LY made critical revision for important intellectual content. All authors contributed to the study conception and design, material preparation, and data collection, read, and approved the final manuscript.

## Conflict of Interest

The authors declare that the research was conducted in the absence of any commercial or financial relationships that could be construed as a potential conflict of interest.

## Publisher’s Note

All claims expressed in this article are solely those of the authors and do not necessarily represent those of their affiliated organizations, or those of the publisher, the editors and the reviewers. Any product that may be evaluated in this article, or claim that may be made by its manufacturer, is not guaranteed or endorsed by the publisher.
